# A Google Trends^™^ Analysis of Bladder Cancer: Determining Awareness Campaign Success, and Patients’ Needs in Clinical Management

**DOI:** 10.31557/APJCP.2021.22.10.3115

**Published:** 2021-10

**Authors:** Aykut Demirci, Berat Cem Özgür

**Affiliations:** 1 *Department of Urology, Aksaray University Training and Research Hospital, Aksaray, Turkey. *; 2 *Department of Urology, Ankara Training and Research Hospital, Ankara, Turkey. *

**Keywords:** Bladder cancer, google trends, awareness month, urinary cytology, transurethral bladder tumour resection

## Abstract

**Objective:**

We aimed to determine the interest and changing trends over time in the diagnosis and treatment of bladder cancer and its awareness campaign by examining the Google Trends application as an indicator of people’s interest globally.

**Methods:**

Using the Google Trends application, we determined the yearly and country-based relative search volumes of the term “bladder tumor” and of the methods used in the diagnosis and treatment of bladder cancer in the period from January 2004 to December 2019. We compared the median relative search volumes found in the period 2004-2011 (Period 1) with those found in the period 2012-2019 (Period 2).

**Results:**

We found that the median relative search volume for bladder cancer decreased in period 2 and this was parallel to the decrease in the incidence rates in North America and Australia (p<0.001). We found that the bladder cancer awareness month did not cause an increase in the online interest (p>0.05). We found that the median relative search volumes of diagnostic cystoscopy and cytology were higher than those of molecular markers and imaging methods in line with guidelines (p<0.001). Also, TURBT was the most sought-term among treatment methods with increasing popularity in the second period (p<0.001).

**Conclusion:**

People use the internet intensively to search for information about bladder cancer. We think that several types of web-based applications such as “Google Trends” can help determine the behavioural patterns and tendencies of bladder cancer patients and affect the clinical decision-making processes, as well as readily determining the impact of cancer awareness campaigns to bring about an increased awareness in the society for the recognition of the importance of an early diagnosis.

## Introduction

Bladder cancer is globally the second leading type of cancer with more than 500,000 new cases and almost up to 200,000 deaths among urological cancers (Ferlay et al., 2018). 

Parallel to the increasingly common use of internet today, most people with bladder cancer use search engines and social media as a resource to look for information about their diseases and about respective diagnostic methods and therapeutic options before they discuss with their physicians (Leveridge, 2016; Corfield et al., 2018). Although the accuracy and quality of the information retrieved from the internet are debatable, it is an important tool used for patient education and support in the treatment and decision-making processes because of the common use of the internet among bladder cancer patients (Lee et al., 2003; Salem et al., 2019). 

Google Trends™ is an application created by Google; which is one of the most commonly used internet search engines today. Google Trends™ can provide statistical data about a specific term by a defined time-interval and geographical region, serving as a resource to obtain epidemiological data and information about screening methods and therapeutic options to be utilized by the scientific literature in the healthcare field (Choi and Varian, 2012; Nuti et al., 2014). Traditional qualitative studies and surveys are mostly used to obtain feedback about patient awareness. However; with the advances in the information technology and the widespread use of the internet worldwide, Google search is increasingly preferred for determining public trends over global or local surveys; which can be time-consuming and costly (Jacobs et al., 2017).As with other cancers, creating awareness in society is important for the early diagnosis and treatment of urogenital cancers. Google Trends™ offers an easy-to-access large database to follow up on the awareness about cancer in the society (Seidl et al., 2018). 

In this study, we aimed to determine the situation and changing trends globally about the online interest in bladder cancer awareness and its diagnosis and treatment by utilizing the Google Trends™ application. 

## Materials and Methods

This cross-sectional study was approved by the Aksaray University ethics committee (No: 2020/08-27). We examined medical keywords used for the diagnosis and treatment of bladder cancer in Google’s search engine in the period from January 2004 to December 2019 by using the Google Trends™ application (https://trends.google.com.tr/trends) (Table 1). 

Google Trends™ provides the relative search volumes (RSV) of keywords used in the search. Also, it provides the distribution of keywords by the country, revealing the geographical region that any specific keyword was searched most commonly. The RSV values are scored in the range from 0 to 100. ‘0’ means that the availability of data is inadequate; whereas, ‘100’ indicates the highest popularity for the respective keyword. Other findings are scored relatively between the upper and lower limits in this range. Google Trends™ allows us to compare the data about the keywords used in the search and categorize the trends of interest relative to the highest point in the graph for a specific geographical region and time. 

In our study, we determined RSV for the keywords used in searches. Furthermore, we categorized our findings into two time-intervals as the period from 2004 to 2011 (Period 1) and from 2012 to 2019 (Period 2) and examined the change in the popularity of subjects by comparing the periodic median RSV of each keywords between these two periods. Also; in order to investigate the effect of bladder cancer awareness month of May on bladder tumour searches on the internet, we determined the median RSV of each year by excluding the median RSV of May of each year. Then, we compared the values we found for each year with the median RSV of May of the respective year.

*Statistical Analysis*

Data were analyzed using the SPSS 20.0 (SPSS, Chicago, IL) program. The data were summarized as median and interquartile ranges when outliers were found distorting the data. The homogeneity of the data was tested with the Shapiro-Wilks test. When the Shapiro-Wilks test yielded a p-value of <0.05, non-parametric tests were used, assuming that the data distribution was nonhomogeneous. When the Shapiro-Wilks test yielded a p-value of ≥ 0.05, the distribution of the data was assumed to be homogeneous and the data were tested with parametric tests. For comparing two independent values; the independent sample t-test was used when the data met the parametric test assumptions, whereas the Mann-Whitney U test was used when the data did not meet the parametric test assumptions. The paired samples t-test was used for comparing two dependent variables meeting the parametric test assumptions. When the parametric test assumptions were not met, the Wilcoxon signed-rank test was used for comparing two dependent variables.

## Results

**Figure 1:**
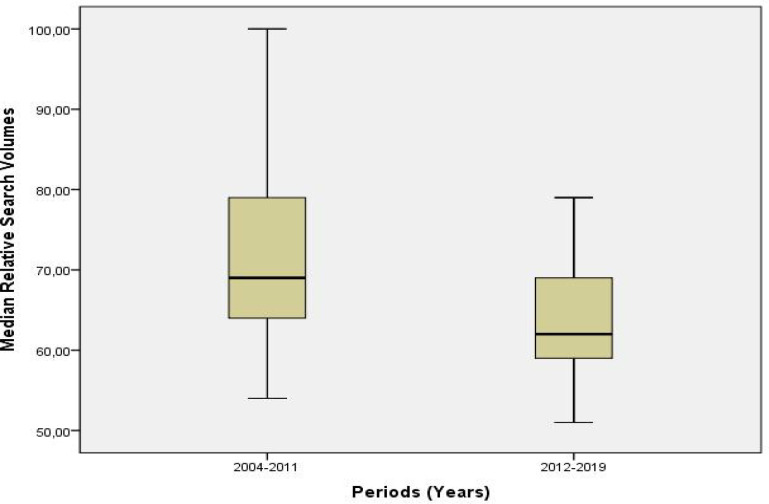
Comparison of the Relative Search Volumes of the “Bladder Cancer” Term on the World Wide Web by Periods

**Figure 2:**
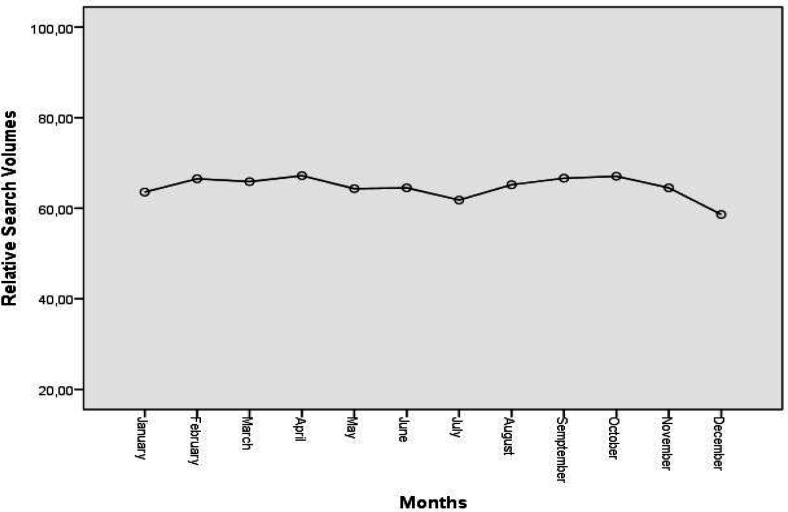
The effect of Bladder Cancer Awareness Campaign on the Median Relative Search Volumes Between 2004-2019

The median RSV for bladder cancer was 69 (15) in period 1 and it was 62 (10) in period 2, pointing out a decline (p <0.001) (Figure 1). The comparison of the data by the geographical regions demonstrated that among the five countries displaying the highest trends of interest in period 1, the population’s interest in the subject declined in the United States, New Zealand, and Australia, and the US was replaced by the United Kingdom in period 2 (Table 2). When we examined data of both the recent 15 years and the comparative data of the two periods, we observed that the median RSV values for bladder cancer were not different in the month of awareness (p> 0.05) (Table 3, Figure 2).

When the search words about molecular markers and cytology were compared as the diagnostic tests, it was observed that the median RSV values for cytology were higher in both periods but the population’s interest declined for both markers and cytology in period 2 (p <0.001, p= 0.014; respectively). The comparison of the interest in imaging methods and cystoscopy revealed that the median RSV values for cystoscopy were higher in both periods, increasing especially in the second period (p <0.001). The RSV values for the urinary bladder ultrasound were stable across both periods (p = 0.41). The median RSV value for computed tomography (CT)-urography increased in period 2 compared to period 1. However, the median RSV value for intravenous pyelography was lower in period 2 compared to period 1 (Table 4). 

The comparison of the modes of treatment options revealed that the mean RSV values for the transurethral resection of the bladder tumour (TUR-BT) were higher than those of radical cystectomy in both periods, increasing further in the second period (p <0.001). Regarding the intravesical therapies, BCG (Bacillus Calmette-Guérin) was more popular in both of these periods compared to chemotherapy; however, the median RSV value for BCG declined in the second period (p = 0.003). The comparison of the most sought keywords associated with urinary diversions demonstrated that ileal conduit was the most popular in both periods and there was not a difference in the median RSV between the two periods (p = 0.27). The median RSV for orthotopic bladder and Indiana pouch displayed a tendency to decline across both periods (p <0.001) (Table 5).

**Table 1:** Bladder Cancer Related Terms Searched Worldwide via the Internet Between 2004-2019

Cancer Type
Bladder Cancer
Diagnostic Terms
NMP22
Urovysion
BTA stat
Urine Cytology
Bladder Ultrasound
CT Urogram
Intravenous Pyelography
Cystoscopy
Treatment Terms
TURBT
Radical Cystectomy
İleal Conduit
Indiana Pouch
Orthotopic Neobladder
Intravesical Chemotherapy
Intravesical BCG

CT, Computed tomography; TURBT, Transurethral resection of bladder tumor; BCG. Bacille Calmette-Guérin; NMP, Nuclear Matrix Protein; BTA, Bladder Tumor Antigen

**Table 2:** Relative Search Volumes of the “Bladder Cancer” Term by Country Between 2004-2019

Countries	RSV
2004-2011 Period	
USA	100
Canada	82
New Zeland	69
UK	69
Australia	66
Ireland	49
2012-2019 Period	
UK	100
USA	89
Canada	85
Australia	65
Ireland	62
New Zeland	60

USA, United States of America; UK, United Kingdom; RSV, Relative search volume

**Table 3:** The Global Impact of the Bladder Cancer Awareness Month on the Relative Search Volumes Between 2004-2019

Periods	Baseline RSV	Awareness Month RSV	p values
2004-2019	62.5(11)	60.5(14)	0.70
2004-2011	65(17.75)	61.5(21.75)	0.68
2012-2019	59(10)	59(11)	0.74

RSV, Relative search volumes; values are reported as median (Interquartile range)

**Table 4:** Comparison of the Global Online Interest for the Diagnostic Tests Between 2004-2019

	Periods		p values
	2004-2011	2012-2019	
	RSV	RSV	
NMP22	25.5 (26.25)	8.5(4)	<0.001
Urovysion	17 (16)	5 (2.75)	<0.001
BTA stat	3 (7)	2(1)	<0.001
Urine Cytology	34.5 (19.75)	34 (8)	0.014
Bladder Ultrasound	3.5 (3)	4(2)	0.41
CT Urogram	3 (1)	4(1)	0.001
Intravenous Pyelography	1 (1)	1(0)	<0.001
Cystoscopy	70 (9)	74 (10.75)	<0.001

Values are reported as median (Interquartile range); RSV, Relative search volumes; CT, Computed tomography; NMP, Nuclear Matrix Protein; BTA, Bladder Tumor Antigen

**Table 5:** Comparison of the Global Online Interest for the Treatment Options Between 2004-2019

	2004-2011	2012-2019	p values
	RSV	RSV	
TURBT	40.5 (9.75)	56 (22.75)	<0.001
Radical Cystectomy	15 (9)	14 (3)	0.14
Ileal Conduit	50.5 (13.75)	51.5 (6.75)	0.27
Indiana Pouch	4 (5)	1.5 (1)	<0.001
Orthotopic Neobladder	15 (7.75)	10 (2)	<0.001
Intravesical Chemotherapy	5 (11.75)	5 (2)	0.28
Intravesical BCG	11 (18.75)	10 (5)	0.003

Values are reported as median(Interquartile range); RSV, Relative search volumes; TURBT, Transurethral resection of bladder tumor; BCG, Bacille Calmette-Guérin

## Discussion

The rise in the use of internet has allowed people to type the keywords of their interest into search engines to look for information about signs and symptoms of diseases, diagnoses, and treatments that they are acknowledged by physicians; that they hear over in their social environment, and that they come across on the social media and throughout their education. Google is the most widely used search engine worldwide and Google Trends™ allows us to look for the trends in the most commonly searched keywords (Nuti et al., 2014; Mavragani and Ochoa, 2019). We utilized this application and investigated the trends of interest in bladder cancer and found out that the population’s interest in this type of cancer has decreased recently. The bladder awareness campaigns held in May of each year all around the world was not found to influence the interest in bladder cancer. Also, the respective search rates showed a dynamic trend by the geographical regions. A recent study examining the worldwide status of bladder cancer reported that the incidence of bladder cancer increased in Canada to some extent but it had decreased in North America and Oceania since the mid-1990s. It was reported that the observed decline resulted from the reduced smoking rates in those countries over the past years (Antoni et al., 2017). A study examining the awareness on the aetiology of bladder cancer found that the majority of people were unaware of the bladder cancer-inducing effects of smoking. Also, it was demonstrated that even patients diagnosed with bladder cancer and receiving therapy for this disease were unaware of the factors causing bladder cancer. Those three studies mentioned above show that the awareness about bladder cancer has not adequately been created across the populations, yet (Westhoff et al., 2016; Mithani et al., 2018). In the current study, we found that the disease incidence, the effects of the awareness months, and changing trends by the geographic regions were parallel to the online interest of the population. To our knowledge, this is the first study investigating the trend of search queries for bladder cancer. Also, we consider that these findings are in agreement with the information in the literature and that Google Trends™ application can be used for epidemiological purposes and campaigns. 

When we examined the interest in searching the methods used in the diagnosis of bladder cancer on the internet, we found that the median RSV for cystoscopy showed an increasing trend in the last eight years and displayed the highest RSV value in both periods compared to other imaging methods. The examination of non-invasive diagnostic methods showed that urinary cytology was associated with higher median RSV values in both periods, compared to the FDA (US Food and Drug Administration) approved tests like NMP22, Urovysion, and BTA stat markers. Moreover, we found that the interest in the molecular markers showed a decreasing trend in the last eight years. European Association for Urology underlines that cystoscopy is the gold standard in patients with suspicious symptoms suggesting bladder cancer and that cytology should be employed in combination in order to detect high-grade tumours (Babjuk et al., 2019). Due to the inadequacies of cytology in diagnosing low-grade tumours and despite the introduction of several highly sensitive urinary markers for early diagnosis as non-invasive methods with ease of application in the routine follow-up practice; only a few of them have been approved by the FDA and no consensus has been reached on their use in the diagnosis of bladder cancer, yet (Schmitz-Dräger et al., 2015; Soria et al., 2019). A study on 64,450 patients in the Surveillance, Epidemiology, and End Results (SEER)-Medicare database reported increased use of markers in parallel to the introduction of several new urinary markers and the popularity of conducting research in this scientific area of interest in the period between the years 2001 and 2011. However, it was also reported that the use of molecular markers had decreased recently due to many factors including the unavailability of guideline recommendations, high costs, requirements for the transportation of test materials, lack of availability of tests in all medical facilities, and difficulties of reimbursement raised by insurance systems (Narayan et al., 2018). Our findings indicate that internet searches are parallel to the trends of interest in the current literature, demonstrating that the Google Trends™ application can be used to determine the status of new diagnostic methods in comparison to gold standard modes of treatment in bladder cancer.

When we examined the interest in imaging methods, we found that the interest in bladder ultrasonography had always been high, also the interest in CT urogram increased gradually especially in the last eight years. Although many new imaging techniques have recently been introduced in the literature as adjuncts to the methods already used for clinical staging, the current guidelines still recommend the use of imaging methods including (CT)-intravenous urography (IVU) and urinary bladder ultrasonography for investigating urinary bladder tumours especially in the presence of urinary symptoms and hematuria (Srivastava et al., 2017; Babjuk et al., 2019). The guidelines recommend the use of CT-urography for investigating high-risk tumours close to ureteral orifices, in parallel to the increasing popularity of this technique in the last 10 years. Furthermore, they state that CT-urography may provide more information compared to IVU and that IVU may be an alternative method when CT is not available (Potenta et al., 2015; Babjuk et al., 2019). We think that urinary bladder ultrasonography was found out to be a focus of interest because it is a technique to be used not only for urinary bladder tumours but for benign prostate hyperplasia, urinary stones including those in the bladder, and neurogenic bladder as well. The increasing trend in the interest in CT-urography demonstrates that current guideline recommendations and advances in technology are reflected in the population’s interest in internet searches, suggesting that Google Trends™ can also be used in studies in order to monitor the use of new diagnostic techniques in medicine.

When the interest of people in searching treatment methods over the internet was investigated, it was found that the leading median RSV of both periods with a recently increasing trend belonged to TUR-BT. The investigation of the keywords about intravesical therapies showed that BCG was the most commonly searched one over the internet. About the most commonly searched keywords for urinary diversions performed in combination with radical cystectomy, we found that the most popular terms were the “ileal conduit”, “Indiana pouch”, and “orthotopic neobladder”. Also, the interest in the ileal conduit was the highest but the median RSV for the other two diversion methods displayed a gradual decline. Treatment of bladder cancer starts with the resection of the tumour tissue in the urinary bladder by utilizing the TUR-BT method. Based on the pathological examination report; a re-TUR is performed. Also, a radical cystectomy can be necessary for the treatment of invasive tumours (Martinez Rodriguez et al., 2017; Babjuk et al., 2019). Intravesical therapies such as immunotherapy (BCG) and chemotherapy are adjunct to endoscopic treatment in non-muscle invasive bladder cancers. BCG therapy replaces chemotherapy as the leading mode of therapy since it reduces the risks of both recurrence and disease progression ( Chou et al., 2017; Woldu et al., 2017). In a review, the ileal conduit was reported as the most preferred diversion type worldwide despite several other diversion methods introduced so far (Noldus et al.2018). Because superficial bladder tumours account for the majority of bladder cancers and because the primary mode of treatment is TUR-BT, we think that the most commonly searched terms were associated with these entities. Also, we observe that; considering the currently known benefit to risk ratio, BCG method is offered to patients more commonly and this is reflected on the internet search trends. We think that the actual increasing trend towards the current diversion types leads to the same increase in online searches. The agreement of our study results with the literature in terms of the current status of the bladder tumour treatment suggests that the Google Trends™ application may be useful in monitoring the trends about current therapeutic approaches. Consequently, both patients and healthcare providers will be provided with benefits, as the former will obtain information about therapeutic options and the latter will have access to a variety of data sets for research. 

Our study has some limitations. While Google Trends™ reflects the population’s interest by providing data about the looked-up information on the Google search engine, it does not cover all types of the quest for information performed on other search engines or in regions with limited or no access to the internet. Furthermore, Google Trends™ has some limitations in itself, as the data to be retrieved from Google Trends™ depend on search terms and the language of the search; the age, gender, and occupation of the users are not accessible, and the quality of the information provided to the users cannot be determined. 

In conclusion, it has been observed that people use the internet widely to obtain information about bladder cancer. By examining the online interest, we determined that the bladder cancer awareness campaign failed to create the intended impact and that the search for information about diagnosis and treatment methods on the internet showed a parallel development to the content of current guidelines. Based on the results of this study and considering Google Trends™ as a measure of the population’s interest; we think that Google Trends™ may be useful for analyzing the behavioural patterns of those seeking information about bladder cancer, for improving clinical practice in alignment with the patient’s needs and training requirements, and for saving money and time in conducting global and local surveys although the application itself needs further improvements in the software.

## Author Contribution Statement

Concept, Study Design, Data Collection, Analysis, Writing, A.D.; Supervision, Critical Review, B.C.Ö.
